# Notes and News

**Published:** 1892-02-13

**Authors:** 


### Notes and News.

Collected and Communicated.

Mr. Henry Irving will preside at the annual dinner of the North London Hospital for Consumption, Hampstead, to be held at the Hotel Métropole on May 28th.

The fourth general meeting of the current session of the Hospitals Association was held on the 4th instant at the Society of Arts, John Street, Adelphi, under the presidency of the Right Hon. the Lord High Chancellor, when Mr. B. Burford Rawlings read a paper on "The Chronic Indigence of our Hospitals." Among those present were Mr. T. Bryant, F.R.C.S. (President of the Royal College of Surgeons), Dr. Bristowe, F.R.S., Dr. Gilbart Smith, Mr. Timothy Holmes, Mr. Gainsford Bruce, M.P., Dr. Steel, and a large number of hospital secretaries and others. As Mr. Rawlings proposes to republish his paper in full, we shall merely indicate a few of the points dealt with. Mr. Rawlings, commencing with the statement that the income of the whele number of the metropolitan hospitals was a diminishing quantity, proceeded to deal with the remuneration of the honorary medical staff, which he was not in favour of; the remuneration of nurses, which he declared to be on a scale scarcely above a housemaid's wages; the expenditure of the hospitals, which he put at £650,000, of which one-third part was supplied by endowments unequally distributed; the deficiency on capital account; the obsolete and unsanitary buildings; the defective provision for convalescents; and the absence of an attempt to adjust the provision to the needs of an increasing population. He forcibly showed that each institution is mainly supported by a small group of special friends, citing, as an instance, a case where eighty-eight persons contributed £46 000 out of a total of £55,000 raised in a given year. He insisted upon the importance of common action, and was certain that sooner or later the hospitals would be brought to work in unison and to fight the enemy under one flag. It was not enough that our hospitals should deserve a competency, but, for the sake of our civilisation, they should be able to demand it.

Mr. T. Bryant, in opening the discussion which followed the reading of the paper, said it was a most grievous thing that they could not record so many givers to hospitals as they did a few years ago. He did not know why that was, unless it was that people felt that they did not quite know why hospitals should be supported. Most of those in that room would probably say they should be supported because of the universal good they were to the poor, but he was not disposed to think that so important as the good they did for the rich as schools of medicine at which hundreds of young men were educated and sent out all over the world.

Dr. Bristowe referred to the value of hospitals as affording opportunities for surgical research, and cited the great progress in modern surgery, with the use of anæsthetics and aseptic methods of treatment, as proof of the value of hospitals in that direction. The improvement in nursing was also due to the hospitals, and he thought that if these facts were brought home to the public it must result in an increase of subscriptions. Mr. T. Holmes advocated the adoption of the scheme for a central hospital authority, as laid before the Lords' Hospital Committee by the C. O. S.

Mr. Burdett said that, briefly put, the object of the meeting was to try to fix public attention on the fact that the hospitals of London had to meet an exceptional deficiency this year—one of £150,000 in round numbers. He was afraid there was a great want of more sympathy both from the public and the Press, this being shown in the latter case by the frequency of the heading "Hospital Scandal," although in almost every case the article which followed concerned a rate-supported institution. If the public would only examine these cases, they would experience a feeling of confidence, and even of thankfulness, when they realised that it was owing to the hospitals that the present high state of voluntary efficiency was due. He was also afraid that the public failed to realise the immense amount of suffering relieved by the hospitals.

Mr. Gainsford Bruce, M.P., expressed a hope that the hospitals would always be dependent upon the free-will offerings of the public. The Lord Chancellor said that the voluntary character of the hospitals seemed to him the essence of their existence. Government aid did mean Government supervision, and would lead to a cessation of voluntary aid, for he never yet heard of any one voluntarily giving anything in addition to the tax which Government demanded. He did not believe his countrymen would be backward in subscribing to the hospitals if only the facts were properly brought home to them, and as every tradesman found it necessary to advertise his wares in every possible way, in order to attract the attention of the busy public, so must the hospitals make their wants known. He could not help being struck by the picture that had been drawn of hospitals suffering, and the statement that for every one admitted two or three had to be turned away. Most strongly did the thought of the rejected ones returning to their homes without hope or medical attendance appeal to one's imagination. Mr. Rawlings having replied to the criticisms on his paper, the proceedings concluded with a vote of thanks to the Chairman and Mr. Rawlings.

The world moves by differences of opinion, and possibly no world has more differences than the hospital world. It is something, however, to disconcert a Lord Chancellor, as Lord Halsbury was disconcerted when presiding at the Hospitals Association meeting on the 4th inst. The reporters sat immediately below the Chairman, and one of their number, who is well known in the gallery of the House of Lords, kept up a running commentary on the views he held and the remarks made by each speaker throughout the course of the addresses. Thus, one speaker heard a voice say, "Hum! Heard that afore;" another, "You are going too fast, and you'll muddle up your sentences;" and a third, "Dull, deadly dull." Each speaker in turn started with the impression that a personal enemy was concealed somewhere in his neighbourhood who was adopting this method of making him appear ridiculous. The truth is, we believe, that the reporter who was the innocent cause of misapprehension to the speakers is deaf enough to be unaware that he utters these sentences in an audible voice. The whole scene was very amusing, when the situation was grasped, and everyone took the interruptions in good part, from the Lord Chancellor downwards. We have no doubt that the noble lords will be amused to hear that a running comment is kept up in the reporters' gallery throughout the whole of the debates in the House of Lords. Won't some gallery-man report the reporter, and so supply some most excellent copy?

